# A Domain Generalization and Residual Network-Based Emotion Recognition from Physiological Signals

**DOI:** 10.34133/cbsystems.0074

**Published:** 2024-02-05

**Authors:** Junnan Li, Jiang Li, Xiaoping Wang, Xin Zhan, Zhigang Zeng

**Affiliations:** ^1^ School of Artificial Intelligence and Automation, Huazhong University of Science and Technology, Wuhan 430074, China.; ^2^ Hubei Key Laboratory of Brain-inspired Intelligent Systems, Huazhong University of Science and Technology, Wuhan 430074, China.; ^3^ Key Laboratory of Image Processing and Intelligent Control (Huazhong University of Science and Technology), Ministry of Education, Wuhan 430074, China.; ^4^ Institute of Artificial Intelligence, Huazhong University of Science and Technology, Wuhan 430074, China.

## Abstract

Emotion recognition from physiological signals (ERPS) has drawn tremendous attention and can be potentially applied to numerous fields. Since physiological signals are nonstationary time series with high sampling frequency, it is challenging to directly extract features from them. Additionally, there are 2 major challenges in ERPS: (a) how to adequately capture the correlations between physiological signals at different times and between different types of physiological signals and (b) how to effectively minimize the negative effect caused by temporal covariate shift (TCS). To tackle these problems, we propose a domain generalization and residual network-based approach for emotion recognition from physiological signals (DGR-ERPS). We first pre-extract time- and frequency-domain features from the original time series to compose a new time series. Then, in order to fully extract the correlation information of different physiological signals, these time series are converted into 3D image data to serve as input for a residual-based feature encoder (RBFE). In addition, we introduce a domain generalization-based technique to mitigate the issue posed by TCS. We have conducted extensive experiments on 2 real-world datasets, and the results indicate that our DGR-ERPS achieves superior performance under both TCS and non-TCS scenarios.

## Introduction

**Fig. 1.**
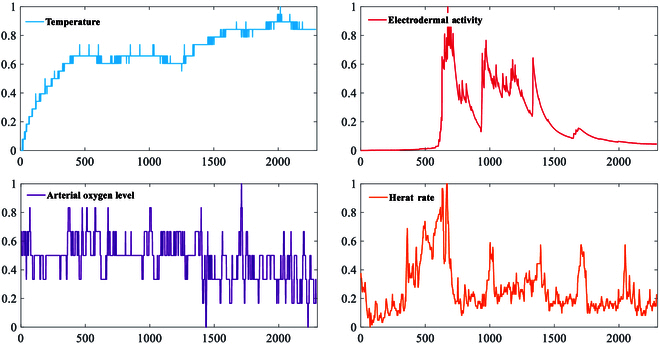
The visualization of several normalized physiological signals in a non-EEG biosignal dataset. Here, the horizontal coordinate is the time (in seconds) and the vertical coordinate is the value of the physiological signal.

Emotion recognition has been widely applied in various fields such as robotics, marketing, education, and industry, due to the rapid increase in the frequency of use of intelligent technology in society and the fast development of related industries in recent years [1]. Human emotions often vary with behavioral, facial, and psychological changes. Hence, many studies have focused on emotion recognition based on facial expressions, verbal texts, audio, and physical behaviors. However, these methods may not accurately reflect human emotions, as people can control their expressions, words, and behaviors through their own subjective consciousness. Emotion recognition from physiological signals (ERPS) is more reliable than the above methods, because most people cannot manipulate their physiological indicators by their subjective willingness. There are many physiological signals currently used for emotion recognition, such as electroencephalography (EEG), electrocardiography (ECG), heart rate variability (HRV), blood volume pulse (BVP), skin temperature (TEMP), and electrodermal activity (EDA) [1,2]. These signals are nonstationary time series, which means that their frequency components are more complicated and that it is more challenging to extract the distinctive patterns from them. Figure 1 shows the visualization of several physiological signals in a non-EEG biosignal dataset [3].

ERPS can be divided into single-signal and multi-signal methods. Theoretically, multi-signal methods hold higher accuracy due to the addition of multiple physiological signals. But it is a tricky problem to simultaneously extract the temporal correlation of the same signal and the spatial correlation between different signals. There are some coping methods for this problem. Oh et al. [4] took the physiological data of each time step as input and abandoned the extraction of temporal features. Kanjo et al. [5] used a sliding window to cut the physiological signal and then viewed it as a single image input to convolutional neural network (CNN) for feature extraction. However, it is difficult to preserve the long-distance temporal correlation due to the limitation of the convolutional local receptive field.

Most of the current studies have randomly split the dataset into training, validation, and test sets. However, this is not the case in practical applications. In the real scenario, we are supposed to use past data to train a model and then utilize that model to classify future data. Since physiological signals are nonstationary time series, splitting the dataset based on chronological order may cause a problem called temporal covariate shift (TCS) [6]. Intuitively, the data distribution of physiological signals from the same individual at different moments can vary greatly. This implies that the training, validation, and test sets fail to satisfy the independent identically distributed assumption. There are some approaches based on transfer learning to mitigate TCS, which are divided into 2 main categories, i.e., domain adaptation (DA)- and domain generalization (DG)-based methods [7]. DA-based methods tend to have better performance when access to the target domain data is allowed. In practice, however, access to the target domain data is often not available, so DG is designed to handle this situation. The goal of DG is to learn a model that performs well for unknown data from one or several related domains [8]. Compared to DA-based methods, DG-based methods have better generalization capabilities but are also more challenging.

In order to address the abovementioned issues, we propose a domain generalization and residual network-based approach for emotion recognition from physiological signals (DGR-ERPS). The proposed DGR-ERPS consists of 4 main components, i.e., feature pre-extraction, 3-dimensional (3D) image data generation, residual-based feature encoder (RBFE), and domain segmentation and alignment. We first split the original time series and extract time- and frequency-domain features from each subseries to compose a new time series for the purpose of reducing computational complexity. These time series are then converted into local and global 3D image data. Finally, in order to capture the correlation between physiological signals at different times and of different types, we construct a feature encoder (i.e., RBFE) based on residual network [27] and treat the 3D image data as the input to this encoder. Furthermore, a DG-based technique is applied to diminish the negative impact caused by TCS, which consists of 2 parts, i.e., domain segmentation based on Kmeans++ [9] and domain alignment based on maximum entropy principle [10].

The main contributions of this paper are summarized as follows:

• We propose an emotion recognition method, DGR-ERPS, which addresses the problems of long series, difficulty in capturing the correlation, and variability in data distribution of physiological signals to some extent.

• The original time series is converted into the input for a dual-stream residual network through the steps of feature pre-extraction and 3D image data generation. The RBFE we constructed can sufficiently extract the correlation information between different physiological signals.

• We introduce a method based on DG to alleviate the TCS problem, which consists of 2 steps: domain segmentation and alignment.

• We conduct multiple experiments on 2 public datasets, WESAD and DEAP, to demonstrate the effectiveness of our proposed DGR-ERPS.

The remaining sections are organized as follows. The “Related Works” section is an introduction to the related works. We describe the proposed approach in detail in the “Methodologies” section. The “Experiments” section provides the experimental results and analysis. The last section (“Conclusion”) summarizes our work.

## Related Works

Emotion recognition has been an important research area, and the rapid advances in sensors and wearables have made it possible to use physiological signals for emotion recognition [11]. Most of the early research on ERPS was based on manually designed features and traditional machine learning classifiers. Lee et al. [12] proposed a hierarchical model based on decision trees for emotion recognition. Soleymani et al. [13] used pattern fusion strategy and support vector machines (SVMs) to classify EEG signals, pupil responses, and gaze distance for emotion recognition. However, traditional methods usually require complex data analysis, which requires a great deal of expert knowledge.

In order to overcome the limitations of traditional methods, deep learning techniques were proposed that allow computational models consisting of multiple processing layers to learn data representations [14]. Some deep learning-based methods mainly design learnable networks from the perspective of feature extraction and multi-source information fusion, and then use the learned features for classification. Lin et al. [15] designed a multimodal-multisensory sequential fusion approach, which applied 4 signals, accelerometer (ACC), BVP, EDA, and SKT, as inputs for the CNN-based model. Yin et al. [16] proposed a multiple fusion layer-based ensemble classifier of stacked autoencoder. Each stacked autoencoder consists of 3 hidden layers to filter the unwanted noise in the physiological signals and derives the stable feature representations. Machot et al. [17] constructed a CNN-based feature extraction network and applied a grid search technique to fine-tune the parameters of that network, and finally, only EDA signals were utilized for recognizing emotions.

Although these methods have achieved better recognition results, they ignored the covariate shift problem when splitting the data, and thus, they have limited generalizability. For this reason, some studies have introduced transfer learning to solve this problem. Zhang et al. [18] proposed a subspace feature elimination method, which solves the covariate shift of EEG by establishing a common space of low-dimensional features between different subjects. Lin [19] proposed a transfer learning scheme based on robust principal components analysis in the presence of less labeled data, achieving a reduction in the feature space gap across intra- and inter-individuals. Li et al. [20] implemented transfer learning between domains by 2 stages. The first stage was to select the source domain samples corresponding to the classification model with the top accuracy ranking by *N* classifiers for learning to avoid the effect of negative migration, and the second stage was to adopt style transfer mapping to reduce the discrepancy between source and target domains. All the above efforts are based on DA, while there are fewer ERPS methods based on DG.

## Methodologies

**Fig. 2.**
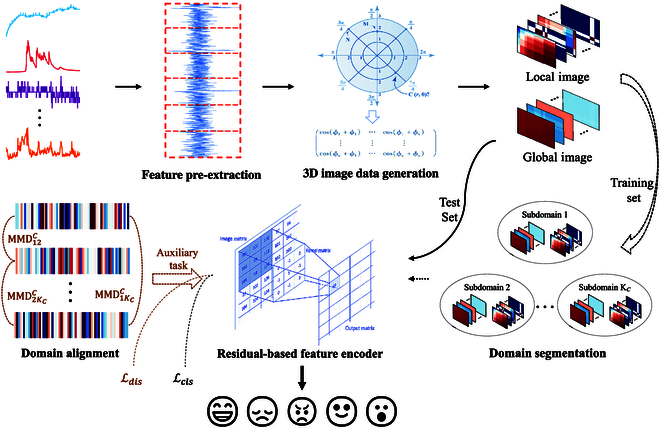
The overall framework of the proposed DGR-ERPS. We pre-extract time- and frequency-domain features from the original time series and transform these series into 3D image data to serve as input for the feature encoder. In the training phase, we utilize the Kmeans++ algorithm to segment the training set into multiple domains and input them into the encoder; these domains are then aligned based on the maximum entropy principle (i.e., auxiliary task), while the emotion recognition is performed (i.e., main task).

We describe our method in detail in this section. As shown in Fig. 2, our DGR-ERPS mainly consists of feature pre-extraction, 3D image data generation, domain segmentation, RBFE, and domain alignment.

### Feature pre-extraction

**Fig. 3.**
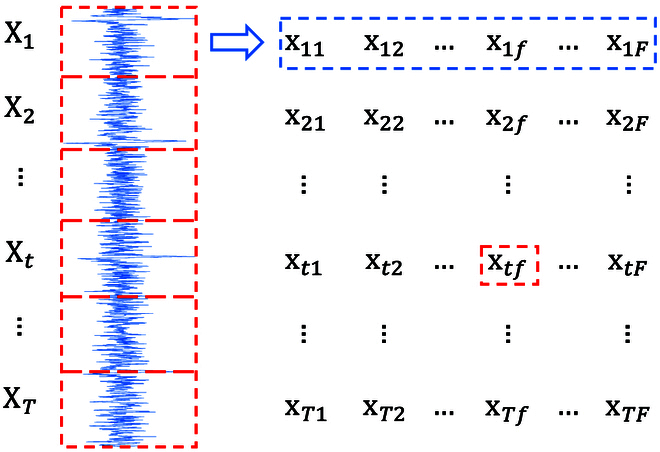
The illustration of feature pre-extraction.

The original physiological signal data typically have a high sampling frequency, e.g., the sampling frequency of the WESAD dataset [21] reaches up to 700 Hz, and that of the DEAP dataset [22] reaches 512 Hz. The higher the sampling frequency, the longer the time series per unit of time, which implies a higher computational complexity. In this work, we first split the original time series into *T* subseries, then extract the time- and frequency-domain features from each subseries, and finally form a new time series of *T* × *F* dimension by composing the extracted features in chronological order. Here, *F* denotes the number of extracted features and *T* denotes the number of subseries. The above process is shown in Fig. 3. Note that x*_tf_* denotes the *f*-th feature in the *t*-th subseries.

In this work, we pre-extract 7 features for each subseries, which include 2 time-domain features and 5 frequency-domain features. The extracted time-domain features are the mean and standard deviation of the values in each subseries, and they can be calculated as:

$$x_{\text{mean}}=\frac{1}{N}\sum_{k=1}^{N}‍x_{k},$$ (1)

$$x_{\mathrm{std}}=\sqrt{\frac{\sum_{k=1}^{N}‍{\left(x_{k}-x_{\text{mean}}\right)}^{2}}{N-1}},$$ (2)

where *x_k_* denotes the *k*-th value in the subseries and *N* denotes the length of the subseries, *k* ∈ {1, 2, ⋯, *N*}. x*_mean_* and x*_std_* denote the mean and standard deviation of the subseries, respectively.

To extract frequency-domain features, we calculate the power spectral density (PSD) [23] of the subseries, transforming it from the time domain to frequency domain. The PSD of the subseries at the *j*-th sampling frequency interval is computed as:

$$\begin{array}{ll} P\left(j\right) & =\frac{1}{N}{\left|\sum_{k=1}^{N}‍x_{k}e^{-i2\pi \frac{\mathrm{jk}}{N}}\right|}^{2}\\ & =\frac{1}{N}{\left|F\left(j\right)\right|}^{2}, \end{array}$$ (3)

where *j* = 1, ⋯, *N*, and *F*(*j*) denotes the discrete Fourier transform (DFT) of *x_k_*. *F*(*j*) can be formalized as:

$$F\left(j\right)=\sum_{k=1}^{N}‍x_{k}e^{-i2\pi \frac{\mathrm{jk}}{N}}.$$ (4)

To further reduce the spectral leakage, the window function is applied to estimate the PSD:

$$\begin{array}{l} P\left(j\right)=\frac{1}{\mathrm{ZN}}{\left|\sum_{k=1}^{N}‍z\left(k\right)x_{k}e^{-i2\pi \frac{\mathrm{jk}}{N}}\right|}^{2},\\ s.t.Z=\frac{1}{N}\sum_{k=1}^{N}‍z{\left(k\right)}^{2}, \end{array}$$ (5)

where *z*(*k*) denotes the window function. In this work, we adopt the Hamming Window [24] as the window function.

On the basis of PSD, we can extract 5 frequency-domain features, which are the spectral entropy, gravity frequency, frequency mean, frequency standard deviation, and root mean square frequency. These features can be computed as follows:

$$x_{\mathrm{se}}=-\sum_{j=0}^{\frac{N}{2}}‍\frac{P\left(j\right)}{\sum_{j=0}^{\frac{N}{2}}‍P\left(j\right)}\log_{2}\frac{P\left(j\right)}{\sum_{j=0}^{\frac{N}{2}}‍P\left(j\right)},$$ (6)

$$x_{\mathrm{gf}}=\frac{\sum_{j=0}^{\frac{N}{2}}‍F\left(j\right)P\left(j\right)}{\sum_{j=0}^{\frac{N}{2}}‍P\left(j\right)},$$ (7)

$$x_{\text{fmean}}=\frac{1}{N}\sum_{j=0}^{\frac{N}{2}}‍P\left(j\right),$$ (8)

$$x_{\text{fstd}}=\sqrt{\frac{\sum_{j=0}^{\frac{N}{2}}‍{\left(F\left(j\right)-x_{\mathrm{gf}}\right)}^{2}P\left(j\right)}{\sum_{j=0}^{\frac{N}{2}}‍P\left(j\right)}},$$ (9)

$$x_{\text{rmsf}}=\sqrt{\frac{\sum_{j=0}^{\frac{N}{2}}‍F{\left(j\right)}^{2}P\left(j\right)}{\sum_{j=0}^{\frac{N}{2}}‍P\left(j\right)}},$$ (10)

where x*_se_*, x*_gf_*, x*_fmean_*, x*_fstd_*, and x*_rmsf_* indicate the spectral entropy, gravity frequency, frequency mean, frequency standard deviation, and root mean square frequency, respectively. Note that *O* types of physiological signals are used in this work, and we take the same feature pre-extraction manner for each type of physiological signal.

### Problem formulation

We denote physiological signals containing *T* times as $X=\left(X_{1},\;X_{2},\;\cdots ,\;X_{T}\right)$, and *T* is also the number of subseries in the feature pre-extraction stage. The physiological signal X*_t_* at time *t* can be represented by the vector (x_*t*1_, x_*t*2_, ⋯, x*_tF_*), X*_t_* ∈ **R***^F^*, and *F* denotes the number of extracted features in the feature pre-extraction phase. Y*_t_* is defined as the corresponding label of X*_t_*. In this work, we utilize a sliding window to cut the time series X to obtain multiple subseries. Assuming that the window size is *W*, the sliding step is 1, and the true emotion corresponding to the last time in the subseries is taken as the label of that subseries, then we get *n* subseries-label pairs {*M_i_*, *y_i_*}. Here, *M_i_* = (X*_i_*, X_*i*+1_, ⋯, X_*i*+*W*−1_), *M_i_* ∈ **R**^*W*×*F*^, *y_i_* = Y_*i*+*W*−1_, and *n* = *T* − *W* + 1.

Note that the above definitions do not take into account multiple types of physiological signals. We adopt the similar definition for each type of physiological signal. Essentially, ERPS is a classification task based on multi-source information in our work. More specifically, a feature extractor *ψ*(·) and classifier *f*(·) are learned to map the input $\left(M_{i}^{1},\;M_{i}^{2},\;\cdots ,\;M_{i}^{O}\right)$ to the corresponding emotion $y_{i}^{\prime }=f\left(\psi \left(M_{i}^{1},\;M_{i}^{2},\;\cdots ,\;M_{i}^{O}\right)\right)$, where *O* is the number of types of physiological signals.

### 3D image data generation

In the realm of time series analysis, long short-term memory (LSTM) [25] and Transformer [26] are broadly adopted because of their powerful semantic relation extraction ability. However, compared with CNNs, they also suffer from some deficiencies. LSTM has difficulty in extracting global information and cannot directly capture the dependent information from long-distance values in time series; Transformer focuses too much on global information and easily ignores the temporal sequential information. Therefore, we design an RBFE to process the time series.

After feature pre-extraction and sliding window processing, each sample *M_i_* contains *F* time series of length *W* (i.e., each feature dimension is regarded as a time series, and there are a total of *F* feature dimensions), where *F* = 7 and *W* is the sliding window size. To satisfy the input requirement of RBFE, we need to convert the multivariate time series into 3D image data. Gramian angular summation field (GASF) [28] can encode univariate time series as image data. A time series in *M_i_* is taken as an example and denotes it as *Q* = (*q*_1_, *q*_2_, ···, *q_j_*), where *j* = 1, 2, ⋯, *W*. In order to make all values fall in the interval [-1, 1], we rescale *Q* by the following equation:

$${\tilde{q}}_{j}=\frac{\left(q_{j}-\max\left(Q\right)\right)+\left(q_{j}-\min\left(Q\right)\right)}{\max\left(Q\right)-\min\left(Q\right)}.$$ (11)

Then, we represent the rescaled time series $\tilde{Q}=\left({\tilde{q}}_{1},\;{\tilde{q}}_{2},\;\cdots ,\;{\tilde{q}}_{j}\right)$ in polar coordinates by encoding the values as the angular cosine and the time as the radius with the following equation:

$$\left\{\begin{array}{cc} \phi_{j}=\arccos\left({\tilde{q}}_{j}\right), & -1\leq {\tilde{q}}_{j}\leq 1,{\tilde{\;q}}_{j}\in \tilde{Q}\\ r=\frac{j}{W},\;j=1,\;2,\;\cdots ,\;W \end{array}\right..$$ (12)

The calculation method of the 2D image *G* is as follows:

$$\begin{array}{ll} G & =\left[\cos\left(\phi_{j}+\phi_{k}\right)\right]\\ & ={\tilde{Q}}^{T}\cdot \tilde{Q}-{\sqrt{I-{\tilde{Q}}^{2}}}^{T}\cdot \sqrt{I-{\tilde{Q}}^{2}}, \end{array}$$ (13)

where *I* is the unit row vector [1, 1, ⋯, 1], *k* = 1, 2, ⋯, *W*, *G* ∈ **R**^*W*×*W*^. It is worth noting that calculating the sum of the inverse cosines (i.e., *ϕ_j_* + *ϕ_k_*) can capture the temporal correlation between the values in *Q*. Through the above steps, we encode the 1D series into the 2D symmetric matrix *G*, which preserves the temporal dependence of the series due to time increases as the position moves from top left to bottom right. Each element *G*_(*j*,*k*| *j*≠*k*)_ of the matrix contains the relationship between the *j*-th and *k*-th time, and each element *G*_*j*,*j*_ is the diagonal element, which contains the value and corner information of the series.

We take the same manner for each time series in *M_i_* in order to convert them into image data and stack them to obtain 3D image data. Here, the size of the 3D image is *W* × *W* × 7. If *O* types of physiological signals are considered, then we can obtain *W* × *W* × 7*O*-dimensional 3D images.

Direct use of Eq. 11 may cause 2 different subseries to have the same scaling result, thus losing global information. For example, scaling the series [1, 2, 3, 4, 5] and [101, 102, 103, 104, 105] both result in [-1, -0.5, 0, 0.5, 1]. We address this problem with the following equation:

$${\tilde{q}}_{i}^{\prime }=\frac{\left(q_{i}-\max\left(\mathbb{Q}\right)\right)+\left(q_{i}-\min\left(\mathbb{Q}\right)\right)}{\max\left(\mathbb{Q}\right)-\min\left(\mathbb{Q}\right)},$$ (14)

where *ℚ* represents the entire time series belonging to the same feature, e.g., (x_11_, x_21_, ⋯, x_*T*1_).

Human physiological signals rarely change in a short period of time. When *max*(*ℚ*) − *min* (*ℚ*) in Eq. 14 is large, some subseries have almost equal values after scaling, leading to the lack of local information. Therefore, in order to preserve both global and local information, we adopt 3D image data obtained from Eqs. 11 and 14 as input for RBFE. Specifically, we mark the local and global 3D images as $G_{\text{local}}$ and $G_{\text{global}}$, respectively.

### Residual-based feature encoder

**Fig. 4.**
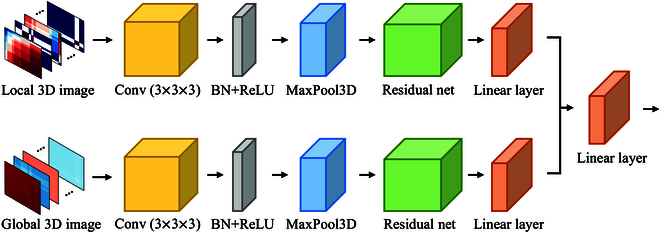
The network structure of RBFE. BN denotes the batch normalization layer.

Inspired by residual network [27], we construct an RBFE, as shown in Fig. 4. RBFE is a dual-stream network whose input is the local 3D image $G_{\text{local}}$ and global 3D image $G_{\text{global}}$. The single-stream network is structured as follows. The input layer consists of a 3D convolution with a convolution kernel size of (3,3,3), a batch normalization layer, a ReLU activation function, and a 3D maximum pooling layer. The input layer is followed by a residual network consisting of some residual blocks, followed by a linear layer. Finally, we concatenate the outputs of 2 single-stream networks and input them into a classifier consisting of a linear layer for emotion classification.

**Fig. 5.**
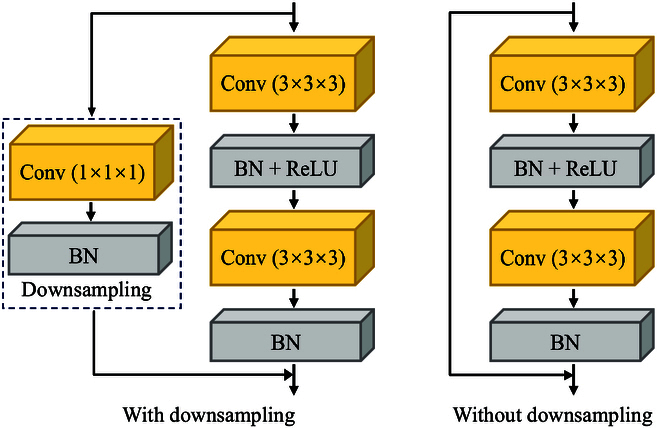
The structure of residual blocks.

There are 2 types of residual blocks, one containing the downsampling layer and the other without the downsampling layer, as shown in Fig. 5. We use 8 residual blocks in this work, out of which the 3rd, 5th, and 7th residual blocks have downsampling layers and the rest of them do not have downsampling layers.

### Domain segmentation and alignment

**Fig. 6.**
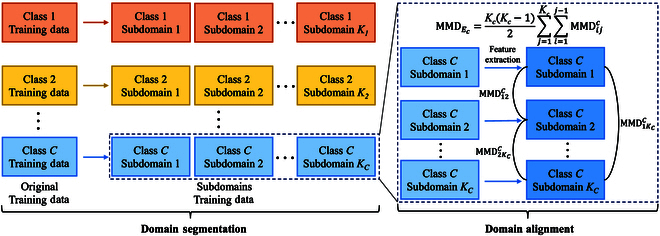
The overall process of domain segmentation and alignment.

In order to alleviate the problem caused by TCS for nonstationary time series, we adopt a DG-based technique for domain segmentation and alignment on the training dataset. First, we perform domain segmentation, i.e., the same emotion samples in the training set are segmented into multiple domains (clusters). Then, we employ the segmented data for model training. In the training phase, in addition to the main task of emotion classification, we also adopt an auxiliary task for domain alignment, i.e., approximating the feature distributions of these domains. Domain segmentation aims to maximize the differences between domains as much as possible, while domain alignment aims to make the model learn a set of network parameters that pull these domains together. Domain segmentation and alignment allows our model to achieve excellent results in the worst case of data distribution, thereby enabling that model to attain decent performance in data with unknown distribution as well. Figure 6 is the overall process of domain segmentation and alignment.

#### Domain segmentation

Physiological signals, unlike data such as images and videos, have no obvious stylistic or background differences to segment the domain. Currently, transfer learning studies based on physiological signals segment the domain by individual. However, these approaches ignore the variability of signals from the same individual and the commonality of signals from different individuals. The variability means that the physiological signals of the same individual may change over time, and the commonality means that the physiological signals of different individuals with the same emotion should be similar.

To address the above issue, we adopt a Kmeans++ -based method for domain segmentation. First, we employ tsfresh (an integrated package for time series feature extraction based on python language; https://github.com/blue-yonder/tsfresh) to extract the statistical features of the samples. After removing the statistical features with dirty data (such as none or infinite), the features with strong relevance are selected using the labels and the Fresh algorithm [29]. Then, we utilize Kmeans++ to cluster the same emotion samples into multiple clusters. To prevent generating clusters that are too small, we sort the clusters by the number of samples, merge the smallest clusters with the closest ones, and update the cluster set. There are 2 distance measures between clusters, and we set 2 thresholds Δ_1_, Δ_2_ to choose which distance measure is adopted. If the cluster size |*D*| < Δ_1_, the center distance of clusters is used as the metric, and if Δ_1_ < |*D*| < Δ_2_, the maximum mean discrepancy (MMD) [30] is used as the distance metric. For 2 clusters *U* = {*u*_1_, *u*_2_, ···, *u_a_*} and *V* = {*v*_1_, *v*_2_, ···, *v_b_*}, the MMD distance is calculated as follows:

$$\begin{array}{c} {\mathrm{MMD}}_{\mathrm{UV}}=\frac{1}{a^{2}}\sum_{i=1}^{a}‍\sum_{i^{\prime }=1}^{a}‍e^{\frac{-{\left\|u_{i}-u_{i}^{\prime }\right\|}^{2}}{2\sigma^{2}}}+\\ \;\frac{1}{b^{2}}\sum_{j=1}^{b}‍\sum_{j^{\prime }=1}^{b}‍e^{\frac{-{\left\|v_{j}-v_{j}^{\prime }\right\|}^{2}}{2\sigma^{2}}}-\frac{2}{\mathrm{ab}}\sum_{i=1}^{a}‍\sum_{j=1}^{b}‍e^{\frac{-{\left\|u_{i}-v_{j}\right\|}^{2}}{2\sigma^{2}}}, \end{array}$$ (15)

where *a* and *b* are the number of samples in *U* and *V*, respectively. *σ* is an artificially selected parameter. The whole process is shown in Algorithm 1. 
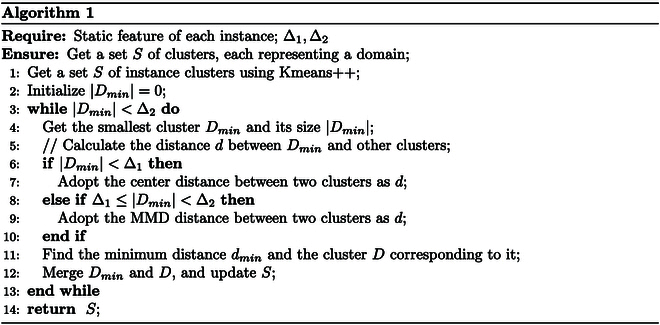


#### Domain alignment

According to maximum entropy principle [10], in order to maximize the utilization of shared knowledge of samples from different distributions, it can be achieved by finding the set of samples that are least similar to each other, which allows a more general and flexible modeling of future data. If a model can learn under the case with the largest distribution difference, then it will have better generalization ability on unseen test data. The above assumption has been validated in the theoretical analysis of time series models [6,31,32]. With domain segmentation, we obtain estimates for the least similar domains, and we next use subdomain alignment to make the model more inclined to extract domain-invariant information.

The role of domain alignment is to make the feature distributions of different domains as consistent as possible, i.e., to make the features extracted by the model more generalizable. Inspired by local maximum mean discrepancy (LMMD) [33], we employ subdomain alignment to align the feature distributions of the same emotion samples from different domains in this paper. We construct a distance loss $L_{\mathrm{dis}}$ for subdomain alignment to minimize the MMD distance of different domains, which is computed as:

$${\mathrm{MMD}}_{E_{c}}=\frac{K_{c}\left(K_{c}-1\right)}{2}\sum_{j=1}^{K_{c}}‍\sum_{i=1}^{j-1}‍{\mathrm{MMD}}_{\mathrm{ij}}^{c},$$ (16)

$$L_{\mathrm{dis}}=\frac{1}{C}\sum_{c}^{C}‍{\mathrm{MMD}}_{E_{c}},$$ (17)

where *K_c_* denotes the number of subdomains of *c*-th class and ${\mathrm{MMD}}_{\mathrm{ij}}^{c}$ denotes the MMD distance between *i*-th and *j*-th subdomains from *c*-th class. The basic idea of $L_{\mathrm{dis}}$ is that feature distributions should be aligned by category: the feature distributions of samples with different emotions should remain different, while those with the same emotions (even if different time and individuals) should remain the same.

The final loss function consists of 3 components, which are classification loss $L_{\mathrm{cls}}$, distance loss $L_{\mathrm{dis}}$, and L2 regularization ∥*W*∥:

$$L=L_{\mathrm{cls}}+\lambda L_{\mathrm{dis}}+\eta ∥W∥,$$ (18)

where *λ* and *η* are trade-off hyperparameters. The cross entropy [34] is used as the classification loss, which is defined as follows:

$$L_{\mathrm{cls}}=-\frac{1}{N}\sum_{i}^{N}‍y_{i}\log\left(y_{i}^{\prime }\right),$$ (19)

where N is the total of samples, *y_i_* denotes the ground-truth label of sample *i*, and $y_{i}^{\prime }$ denotes the predicted probability.

## Experiments

### Datasets

We conduct a number of experiments to validate the performance of our model on 2 datasets, WESAD [21] and DEAP [22], both of which are time series data based on physiological signals.

WESAD contains 6 types of physiological signals for a total of 8 channels, which are BVP (64 Hz), electrocardiogram (ECG, 700 Hz), EDA (700 Hz, 4 Hz), electromyogram (EMG; 700 Hz), respiration (RESP; 700 Hz), and TEMP (700 Hz, 4 Hz). These data come from a chest sensor and a wrist sensor. The dataset collects physiological signals from 15 healthy adults in 4 emotional states (neutral, stress, amusement, and meditation).

DEAP contains 32 channels of EEG signals and 8 channels of peripheral physiological signals. In this paper, only peripheral physiological signals are used, which are 2 channels of electrooculography (EOG), 2 channels of EMG, and one channel each of EDA, RESP, plethysmograph (PG), and TEMP, all sampled at 128 Hz. There are 2 commonly used emotion labels in this dataset, which are valence and arousal, both with values ranging from 1 to 9 points.

### Settings

We train our model on NVIDIA GeForce RTX 3090 GPU. The Adam optimization is used to minimize the loss function. The dropout rate is 0.2, and the batch size is 1,024. For WESAD, the learning rate is set to 8.5 × 10^4^. *λ* is 1.46 × 10^−5^, *η* is 1.15 × 10^−6^, and the window size is 6. For DEAP, the learning rate is set to 6.4 × 10^3^. *λ* is 1.05 × 10^4^, *η* is 2.79 × 10^−6^, and the window size is 11. The threshold values (Δ′ and Δ) are 50 and 300, respectively. The model parameters are initialized using the Kaiming distribution [35]. We use the accuracy and F1 score as evaluation metrics.

### Baselines

On the WESAD dataset, we adopt 5 recent models for comparison. LDA [21] was a traditional machine learning method used by Schmidt et al. when they proposed the WESAD dataset. In that work, they adopted Chest signal for 2- and 3-class emotion recognition. StressNAS [36] was an optimized deep neural network training scheme for neural network architecture search, which decreased the structural design complexity. MMSF [37] was a model that focused on signal fusion, which tended to obtain better performance in the emotion recognition task. ADFES [38] was a decision-level feature fusion model based on traditional machine algorithm. It also achieved good performance due to the inclusion of multiple physiological signals. Self-supervised CNN [39] was a self-supervised deep multi-task learning framework with electrocardiogram representation and emotion recognition as the learning objective, utilizing the common features of 2 tasks to enhance the performance of each task.

On the DEAP dataset, we employ 6 comparative models and record results for both arousal and valence dimensions. MSCNN [40] was a multi-scale CNN model, which decomposed EDA signals into multi-scale views by adopting a coarse-grained method to capture robust and complementary features. RTCAG-1D [41] was an end-to-end multimodal framework, which utilized channel-time attention mechanism to mine dynamic and stable features on time and channels. ERDL [42] fused graph convolutional network and LSTM network, and constructed EEG signals into 3D graphs through differential entropy. Alexnet-2D [41] was an end-to-end image processing network, which converted physiological signals into images for emotion classification. AsMap+CNN [43] captured the asymmetry of different brain regions in 2D vector and then adopted CNN to extract features for emotion classification. MTCA-CapsNet [44] was a new multi-task learning method based on capsule network and attention mechanism, which learns features by utilizing the commonality and difference of different tasks.

### Comparative results

We split the dataset chronologically with a ratio of 8:1:1 for the training, validation, and test sets. The purpose of splitting the dataset in chronological order is to preserve the TCS problem of the dataset. Since there are few studies that take into account TCS, none of the models compared to ours consider the effect of TCS, i.e., these comparative models randomly split the dataset. For the purpose of comparison, we also perform dataset splitting without considering TCS. Tables 1 and 2 display the comparative performance of recent methods on the WESAD and DEAP datasets, respectively.

**Table 1.** The comparative performance of recent methods on WESAD dataset. WS and CN denote the window size (in seconds) and number of classes, respectively.

Model	Signal	WS (s)	CN	Accuracy (%)	F1 score (%)
LDA [21]	Chest signals	0.25	2	91.5	93.1
			3	79.0	74.0
StressNAS [36]	ACC, EDA, BVP, TEMP	60	2	93.1	-
			3	83.4	-
MMSF [37]	Chest and wrist signals	1	3	85.0	86.0
ADFES [38]	ECG, EDA, EMG, BVP	720	3	87.4	86.9
Self-Supervised CNN [39]	ECG	10	4	96.9	96.3
DGR-ERPS (non-TCS)	All signals for non-ACC	6	4	98.1	97.9
DGR-ERPS (TCS)				94.7	94.0

**Table 2.** The comparative performance of recent models on DEAP dataset. WS denotes the window size (in seconds).

Model	Signal	WS (s)	Valence (%)		Arousal (%)	
			Accuracy	F1 score	Accuracy	F1 score
MSCNN [40]	EDA	30	71.4	-	69.3	-
RTCAG-1D [41]	EDA	60	77.2	79.2	83.4	83.8
ERDL [42]	EEG	6	84.8	86.2	85.3	87.1
Alexnet-2D [41]	All signals	10	87.3	78.2	85.5	80.1
AsMap+CNN [43]	EEG	3	95.5	-	95.2	-
MTCA-CapsNet [44]	EEG	1	97.2	-	97.4	-
DGR-ERPS (non-TCS)	All signals for non-EEG	11	99.1	99.0	99.1	99.1
DGR-ERPS (TCS)			80.5	80.0	80.8	79.9

Our DGR-ERPS converts time series into 3D images while considering the TCS problem. Overall, the proposed DGR-ERPS achieves excellent performance in both non-TCS (split the dataset randomly) and TCS (split the dataset chronologically) cases. Under the non-TCS case, DGR-ERPS achieves optimal results on these 2 datasets relative to recent methods. On the WESAD dataset, the accuracy and F1 score reach 98.1% and 97.9%, respectively; on the DEAP dataset, the accuracy of both valence and arousal classifications is 99.1%, and the F1 scores are 99.0% and 99.1%, respectively. These experimental results indicate that the proposed DGR-ERPS has excellent emotion recognition ability. It is worth noting that the performance of our method decreases dramatically in the TCS environment. The 2 performance metrics on the WESAD dataset drop to 94.7% and 94.0%, respectively. The degradation on the DEAP dataset is even more severe, with the accuracy dropping by almost 20%, which indicates that TCS has a very serious negative impact on the model performance.

### Ablation studies

We conduct ablation studies for 4 different settings, whose main goal is to explore the effectiveness of feature pre-extraction, dual-stream network structure, and domain alignment.

• Without feature pre-extraction (w/o FPE). We only use the time-domain mean (i.e., Eq. 1) to process the time series without pre-extracting other features.

• Without dual-stream structure (w/o DSS). We do not input the local image data while removing the corresponding subnetwork, and only the relevant part of the global image data is retained. Note that since emotion recognition using only the local image data is poor, it is not discussed separately.

• Without domain alignment (w/o DoA). We remove the distance loss $L_{\mathrm{dis}}$ in Eq. 18 during training the network.

• Without all modules (w/o All). The 3 modules above are removed at the same time.

**Fig. 7.**
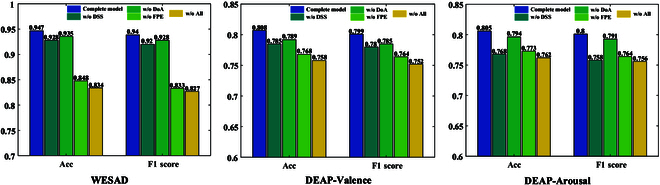
The results of ablation study on WESAD and DEAP.

Figure 7 shows the results of ablation experiments on the WESAD and DEAP datasets. For the WESAD dataset, DSS and DoA make minor improvements to the model. Relative to the complete model, the accuracy and F1 score decrease by 1.9% and 2%, respectively, after removing DSS, and both the accuracy and F1 score drop by 1.2% after removing DoA. FPE contributes more to the model performance, with the accuracy and F1 score of the model decreasing by 9.9% and 10.7%, respectively, after removing FPE. When DSS, DoA, and FPE are all removed, the accuracy and F1 score of the model drop by 10.3% and 11.3%, respectively. For the DEAP dataset, the contribution of FPE to the model is reduced compared to the WESAD dataset. But it is still very crucial, resulting in 3.9%/3.7% and 3.3%/3.7% improvements in the accuracies/F1 scores of the model on valence and arousal classifications, respectively. DSS becomes more crucial for arousal classification on the DEAP dataset compared to the WESAD dataset, increasing the accuracy and F1 score of the model by 3.7% and 4.2%, respectively. DoA still provides limited improvements to the performance, increasing the model’s accuracies/F1 scores by 1.9%/1.4% and 1.1%/0.9%, respectively. After removing the 3 modules mentioned above, the model still performs the worst, with the accuracy/F1 score of valence classification decreasing by 4%/4.7%, and that of arousal classification decreasing by 4.3%/4.4%.

From these ablation results, we can see that the same module has different performance on different datasets. For the DSS module, the improvement on the WESAD dataset is small, while the improvement on the DEAP dataset is large. We believe that this may be due to the larger information content of WESAD data, which dilutes the useful information of local images. After removing DSS, the accuracy/F1 score on the WESAD dataset is much higher than that on the DEAP dataset; however, it is a 4-classification task on the WESAD dataset, which is more difficult than the binary classification task on the DEAP dataset. The FPE module is of importance on both datasets, suggesting that frequency-domain features of physiological signals are essential for emotion recognition.

### Impact of TCS

**Fig. 8.**
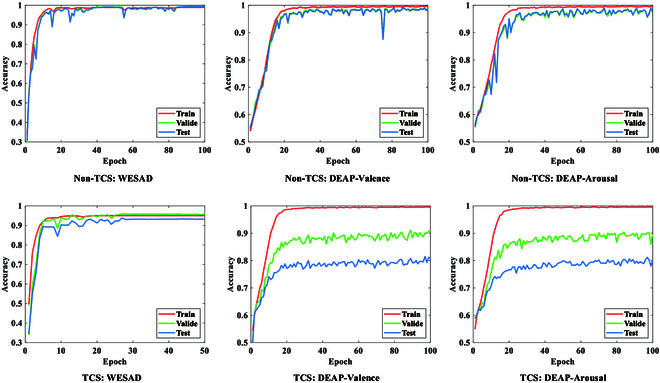
Accuracy variations under non-TCS and TCS cases.

Figure 8 shows the accuracy curves of the training set, validation set, and test set with epoch under both non-TCS and TCS cases. As can be seen, under the non-TCS case, there is a high degree of overlap among these 3 curves on both the WESAD and DEAP datasets. This indicates that the model has no overfitting phenomenon (or the overfitting degree is very low), and it also proves that the proposed DGR-ERPS has a strong emotion classification ability. Under the TCS case, on the other hand, there are different degrees of overfitting phenomena on both the WESAD and DEAP datasets. The reason for this phenomenon is that the presence of TCS causes the distribution of these 3 sample sets (training, validation, and test sets) to be inconsistent. In addition, it can also be observed from the figure that the overfitting is larger on the DEAP dataset, which indicates that the TCS problem is more severe on the DEAP dataset.

**Fig. 9.**
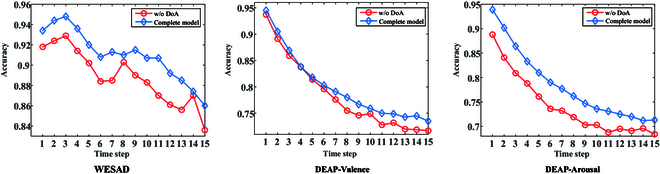
Recognition results under TCS case.

To further analyze the effect of TCS on the recognition results, we split the dataset into 16 parts in chronological order, where the first part is the training set and the last 15 parts are the test sets, and these test sets are sequentially marked as time steps 1 to 15. We compare the performance of the complete model with that of the model after removing $L_{\mathrm{dis}}$ (w/o DoA), and the results are shown in Fig. 9. Overall, on the 2 datasets, the recognition accuracy decreases with increasing time steps, and the rate of decrease becomes gradually smaller. This indicates that the distribution differences between the test set and training set gradually increase with time. It can also be noted from the figure that the blue curve is above or close to the red one, which proves that the DoA module can mitigate the negative impact of TCS to a certain extent.

### Analysis of confusion matrices

**Fig. 10.**
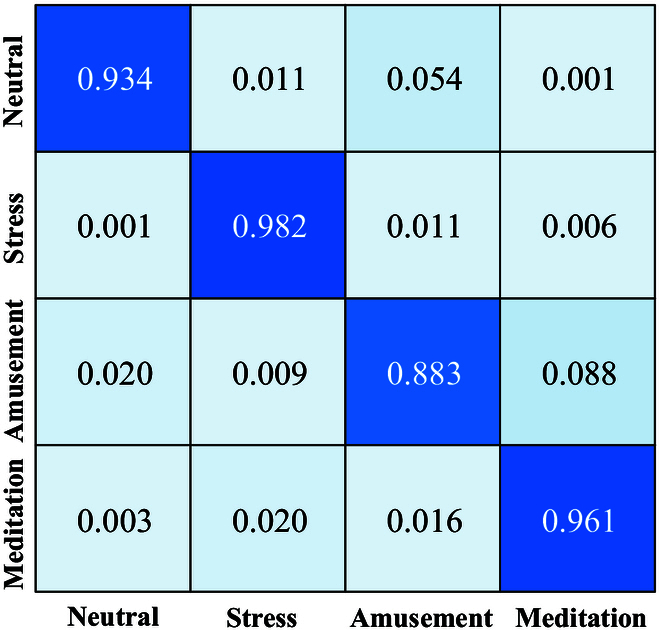
The confusion matrix of WESAD.

We analyze confusion matrices on both datasets to reveal specific classification results for different emotions. Figure 10 shows the confusion matrix on the WESAD dataset. The emotion class with the highest misclassification rate on the WESAD dataset is Amusement, where 8.8% of the Amusement samples are misclassified as Meditation. The emotion class with the lowest misclassification rate is Stress, with a recognition accuracy of 98.2%.

**Fig. 11.**
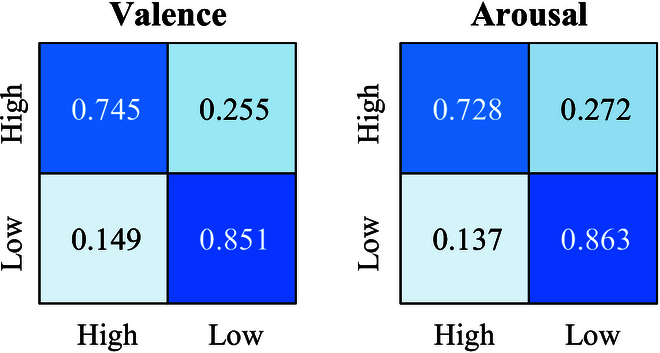
The confusion matrix of DEAP.

Figure  11shows the confusion matrix on the DEAP dataset. On the DEAP dataset, the recognition accuracies of both High Valence and High Arousal are inferior to those of Low Valence and Low Arousal. In addition, the overall accuracy on the DEAP dataset is lower than that on the WESAD dataset, which may be attributed to the different ways of obtaining labels in the 2 datasets. The labels of the DEAP dataset are based on human ratings, which are more subjective and less reliable. The labels of the WESAD dataset are derived from more objective and accurate measures. By analyzing the classification results of different emotions, we can identify which emotions are more challenging to classify, and conduct focused research on them in future work.

## Conclusion

To tackle the dilemma faced by ERPS, we propose a method based on DG and residual network called DGR-ERPS. We split long time series and pre-extract time- and frequency-domain features from the subseries for the purpose of reducing the computational complexity. To fully capture the correlation of different physiological signals, we convert these time series into local and global 3D image data. Meanwhile, we draw inspiration from residual networks to construct an RBFE. Compared to existing ERPS methods, DGR-ERPS considers the nonstationarity of physiological signals, i.e., the TCS problem. For this purpose, we introduce a technique based on DG, which achieves domain segmentation and domain alignment with the help of Kmeans++ and maximum entropy principle, respectively. We perform substantial experiments on 2 datasets based on physiological signals, and the results reveal that DGR-ERPS outperforms previous methods while validating its effectiveness. Our proposed method is a general framework based on multivariate time series and can be easily extended to other time series tasks with nonphysiological signals.

Facial expression itself belongs to image data. In future work, we will explore the fusion of facial expression and physiological signals for multimodal emotion recognition. Meanwhile, due to the heterogeneity gap problem of multimodal data, we focus on the unified framework applicable to multiple modal signals.

## Data Availability

The data used to support the findings of this study are available from the corresponding author upon request.
